# Spleen Tyrosine Kinase Inhibition Ameliorates Tubular Inflammation in IgA Nephropathy

**DOI:** 10.3389/fphys.2021.650888

**Published:** 2021-03-15

**Authors:** Wai Han Yiu, Kam Wa Chan, Loretta Y. Y. Chan, Joseph C. K. Leung, Kar Neng Lai, Sydney C. W. Tang

**Affiliations:** Department of Medicine, The University of Hong Kong, Queen Mary Hospital, Pokfulam, Hong Kong

**Keywords:** IgA nephropathy, spleen tyrosine kinase, inflammation, NF-κB, MAPK

## Abstract

Spleen tyrosine kinase (Syk) is a non-receptor tyrosine kinase involved in signal transduction in a variety of immune responses. It has been demonstrated that Syk plays a pathogenic role in orchestrating inflammatory responses and cell proliferation in human mesangial cells (HMC) in IgA nephropathy (IgAN). However, whether Syk is involved in tubular damage in IgAN remains unknown. Using human kidney biopsy specimens, we found that Syk was activated in renal tubules of biopsy-proven IgAN patients with an increase in total and phosphorylated levels compared to that from healthy control subjects. *In vitro*, cultured proximal tubular epithelial cells (PTECs) were stimulated with conditioned medium prepared from human mesangial cells incubated with polymeric IgA (IgA-HMC) from patients with IgAN or healthy control. Induction of IL-6, IL-8, and ICAM-1 synthesis from cultured PTECs incubated with IgA-HMC conditioned medium was significantly suppressed by treatment with the Syk inhibitor R406 compared to that from healthy control. Furthermore, R406 downregulated expression of phosphorylated p65 NF-κB and p-42/p-44 MAPK, and attenuated TNF-α-induced cytokine production in PTECs. Taken together, our findings suggest that Syk mediates IgA-HMC conditioned medium-induced inflammation in tubular cells *via* activation of NF-κB and p-42/p-44 MAPK signaling. Inhibition of Syk may be a potential therapeutic approach for tubulointerstitial injury in IgAN.

## Introduction

IgA nephropathy (IgAN) was first described over 50 years ago (Tang, 2018) and remains the most common form of primary glomerulonephritis worldwide with a hallmark feature of IgA1 deposits in the glomerular mesangium (Wyatt and Julian, 2013). Most patients with IgAN present a slowly progressive clinical course and about 30–40% of patients will develop kidney failure within 20–30 years of diagnosis (Lai et al., 2016). Apart from a prediction tool that takes into account clinicopathologic features at the time of kidney biopsy (Barbour et al., 2019), there are no other means that take into account pathogenetic processes that accurately predict who will progress to kidney failure. Studies over the past decades have indicated that circulating glycosylated IgA1 binds to autoantibodies and forms immune complexes that deposit in the mesangium, resulting in activated resident cells and local inflammation (Novak et al., 2018). More evidence have shown that mesangial-derived mediators contribute to the pathogenesis of tubulointerstitial damage and podocyte injury *via* mesangial–podocytic–tubular crosstalk (Leung et al., 2018). Genome-wide association studies have also demonstrated that genetic components are implicated in disease pathogenesis and identified several susceptibility genes and loci associated with immune regulation (Zhang et al., 2017). Although important progress has been made in understanding the pathogenic mechanism of IgAN since the disease was first discovered, an effective and specific therapy for IgAN is still lacking (Barratt and Tang, 2018). Tampering the immune system stems from partial treatment efficacy using various forms of immunosuppression such as corticosteroid (Lv et al., 2017), mycophenolate (Tang et al., 2010), and more recently hydroxychloroquine (Liu et al., 2019).

Spleen tyrosine kinase (Syk) is a cytoplasmic tyrosine kinase highly expressed in most immune cells, where Syk plays a critical role in cell signaling during hematopoietic cell activation and differentiation (Mocsai et al., 2010). Syk is activated by stimulation of immunoreceptor expressed on immune cells, the two SH2 domains of Syk specifically bind to the dual phosphorylated immunoreceptor tyrosine-based activation motifs (ITAMS), triggering kinase activation and multiple downstream signaling pathways (Kaur et al., 2013). Syk is also expressed in various non-hematopoietic cells including epithelial cells, endothelial cells, fibroblasts, and neuronal cells. Numerous studies have revealed a diverse biological role of Syk in cell adhesion, platelet activation, vascular development, and cancer growth (Yanagi et al., 2001; Bartaula-Brevik et al., 2018). Given that Syk is an upstream mediator of multiple signaling pathways in the immune responses, it has been used as a potential therapeutic target for autoimmune diseases and immune-mediated disorders (Ghosh and Tsokos, 2010; Lucas and Tan, 2014; Szilveszter et al., 2019).

Syk is also expressed in human and murine mesangial cells and plays a pathogenetic role in IgAN. Syk expression is required for the IgA-induced production of inflammatory cytokines including MCP-1, IL-6, IL-8, and RANTES in human mesangial cells (Kim et al., 2012). IgA may bind to a novel Fcα receptor that mediates phosphorylation of Syk and MCP-1 synthesis in IgA-activated mesangial cells (Barratt et al., 2000; Tsuge et al., 2003), though the expression of Fcα receptor on human mesangial cells is controversial (Leung et al., 2000). Other potential IgA receptor present on mesangial cells such as transferrin receptor (CD71) and galactosyltransferase 1 have been identified to recruit Syk for activation (Moura et al., 2001; Molyneux et al., 2017). *In vivo*, treatment with fostamatinib, the selective Syk inhibitor and a prodrug of R406, significantly reduces proteinuria, glomerular macrophage infiltration, and tissue damage in an animal model of antibody-mediated glomerulonephritis, suggesting a potential protective effect of Syk inhibition on IgAN (Smith et al., 2010).

In this study, we report that Syk is an important mediator of tubular activation in IgAN. Both expression and phosphorylation of Syk were upregulated in renal tubules of kidney biopsies from patients with IgAN. We also found that inhibition of Syk could attenuate the inflammatory changes in PTECs triggered by glomerulotubular crosstalk, *via* downregulation of NF-κB and p-42/p-44 MAPK signaling.

## Materials and Methods

### Sample Collection

Serum samples were collected from Chinese patients (age 49 ± 12 years) with clinical (eGFR 60.52 ± 25.04 ml/min per 1.73 m^2^ and serum creatinine 169 ± 130 μmol/l) and renal immunopathological diagnosis of primary IgAN (*n* = 20) and healthy subjects (*n* = 20) with no microscopic hematuria or proteinuria as normal controls. Renal tissue biopsy were obtained from patients with IgA nephropathy (*n* = 5). Normal portions of renal tissues removed from nephrectomy specimens for the treatment of solitary renal carcinoma in the opposite pole were used as control (*n* = 5). Table 1 provides the clinical characteristics of the five patients with IgAN at the time of biopsy. This study was conducted in accordance with the principles of the Declaration of Helsinki. The use of serum and tissue specimens for this study was approved by the Research Ethics Committee/Institutional Review Board of the University of Hong Kong/Hospital Authority Hong Kong West Cluster. Written informed consent was obtained from all subjects before sample collection.

**Table 1:** Clinical characteristics of patients with IgAN at the time of biopsy.

Pt ID	Sex	Age (years)	sCr (μmol/L)	eGFR (ml/min per 1.73 m^2^)	UPCR (mg/mmol Cr)	Hypertension (Y/N)	On RAAS blocker (Y/N)	On Corticosteroid (Y/N)
1	M	71	92	70	77	Y	Y	N
2	F	58	81	63	189	Y	Y	N
3	F	28	202	26	113	N	Y	N
4	M	60	82	83	540	N	N	N
5	F	54	73	72	99	N	Y	N

eGFR, estimated glomerular filtration rate; IgAN, IgA nephropathy; Pt, patient; RAAS, Renin-angiotensin-aldosterone system; sCr, serum creatinine; UPCR, spot urine protein-to-creatinine ratio.

### Isolation of IgA1

Polymeric IgA1 was isolated and purified from sera of IgAN patients and healthy subjects as described previously (Leung et al., 2001). Briefly, IgA1 was purified using a jacalin-agarose affinity column from Pierce (Rockford, IL, United States), and the purity was confirmed by SDS-PAGE. Five groups of pooled IgA, each from four different IgAN patients or healthy control subjects, were used in the subsequent experiment.

### Immunohistochemical Staining

Kidney biopsies were fixed in 10% neutral-buffered formalin and paraffin-embedded. Immunohistochemical staining was performed on tissue sections (4 μm) by incubation with primary antibody against total Syk and phosphor-Syk (Cell Signaling Technology, Beverly, MA), followed by peroxidase conjugated secondary antibodies. All sections were counterstained with hematoxylin. The percentage of stained area was quantified by Image J software (NIH).

### Cell Culture

Normal human mesangial cells (HMCs) were obtained from Lonza (Walkersville, MD, United States) and grown in RPMI 1640 medium supplemented with 5% fetal bovine serum (FBS; Invitrogen, Carlsbad, CA, United States) at 37°C in 5% CO_2_ atmosphere. Human primary renal proximal tubular epithelial cells (PTECs) were obtained from Lonza and cultured in renal epithelial cell growth basal medium (REBM) with growth supplements at 37°C in 5% CO_2_ atmosphere. In all experiments, cells grown between passages 3–6 were used and serum starved for 24 h before stimulation.

### Preparation of IgA HMC Conditioned Media and Stimulation of PTECs

Conditioned media (IgA HMC medium) were prepared by incubating HMCs (1 × 10^6^ cells in a six-well culture plate) with pooled IgA (50 μg/ml) from IgAN patients or healthy control in RPMI 1640 medium containing 0.5% FBS for 48 h. Cells incubated with RPMI medium alone were used as the medium control. The supernatant of cell culture was collected and stored at −70°C until use. Confluent, growth-arrested PTECs were incubated in RPMI 1640 medium containing 0.5% FBS and 20-fold diluted conditioned medium for 4 h (gene analysis) and 48 h (protein analysis) with or without pre-treatment of Syk inhibitor R406 (2 μM) for 1 h. PTECs were incubated with TNF-α (10 ng/ml) for 48 h.

### RNA Extraction and Real-Time PCR Analysis

Total RNAs were isolated from PTECs using Trizol reagent (Invitrogen). One micrograms of total RNAs were reverse transcribed to cDNA and mRNA expression were detected by ABI Real-time PCR System using SYBR Green Master Mix (Applied Biosystems, Carlsbad, CA, United States). Primer sequences were IL-6, forward 5'-ATGAACTCCTTCTCCACAAG-3' and reverse 5'-TGTCAATTCGTTCTGAAGAG-3'; IL-8, forward 5'-GTGCAGTTTTGCCAAGGAGT-3' and reverse 5'-TAATTTCTGTGTTGGCGCAG-3'; ICAM-1, 5'-GGCCTCAGTCAGTGTGA-3' and reverse 5'-AACCCCATTCAGCGTCA-3'; β-actin, forward 5'-TGACGTGGACATCCGCAAAG-3' and reverse 5'-CTGGAAGGTGGACAGCGAGG-3'. Relative gene expression was obtained after normalization with β-actin, and followed by comparison to control group using SDS software (Applied Biosystems).

### Cytokine Detection by ELISA

Culture media were collected after 48 h incubation. IL-6, IL-8, and ICAM-1 protein levels were quantified using commercial kit (PeproTech, Rocky Hill, NJ) according to manufacturer’s instructions. The detection sensitivity range is 24–1,500 pg/ml for IL-6, 8–1,000 pg/ml for IL-8, and 23–3,000 pg/ml for ICAM-1.

### Western Blot Analysis

Cells were lysed with lysis buffer containing protease inhibitor cocktails (Sigma-Aldrich, St. Louis, MO). Equal amounts of protein were resolved in 12% SDS-PAGE gel (Invitrogen) and transferred to PVDF membrane (Millipore, Bedford, MA, United States). After blocking, the membranes were incubated with antibodies against phosphor-NF-κB (p-p65), NF-κB (p65), phosphor-p42/p-p44 MAPK (p-p42/p-p44), and total p42/p44 MAPK (Cell Signaling Technology), and subsequently incubated with peroxidase-conjugated secondary antibody (Dako, Carpinteria, CA, United States). The immunocomplex was visualized with ECL prime chemiluminescence (GE Healthcare, Buckinghamshire, United Kingdom) using the ChemiDoc XRS+ system (Bio-Rad, Hercules, CA, United States). Quantification of protein bands was performed by the ImageJ program (NIH, Bethesda, MD, United States).

### Statistical Analysis

All the data were obtained from at least three independent experiments and expressed as mean ± SEM. Differences between multiple groups were evaluated with one-way analysis of variance followed by Bonferroni’s comparison using GraphPad Prism, version 4 (GraphPad Software, San Diego, CA, United States). Data were considered statistically significant at *p* < 0.05 (^∗^*p* < 0.05; ^∗∗^*p* < 0.01, and ^∗∗∗^*p* < 0.001).

## Results

### Increased Expression of Total and Phosphorylated Syk in Renal Biopsies From Patients With IgAN

**Figure 1:**
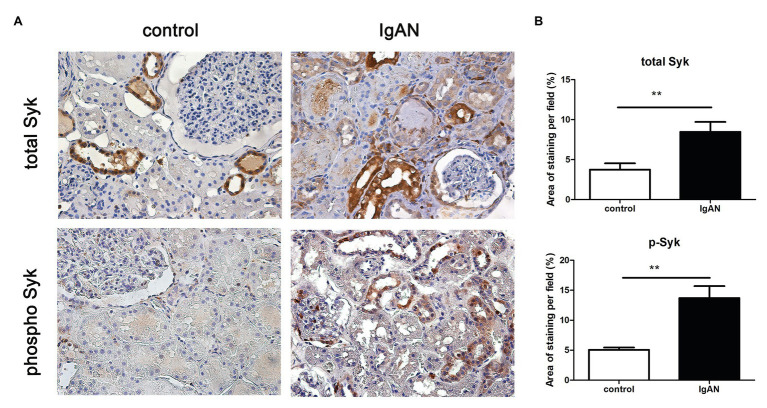
Spleen tyrosine kinase (Syk) expression in human kidney tissues. **(A)** Total and phosphor Syk protein were detected renal biopsies from normal control and patients with IgA nephropathy (IgAN) using immunohistochemical staining. **(B)** Quantitative analysis of different groups. ^**^p < 0.01 between groups as indicated. 400X magnification.

To confirm the activation of Syk signaling in IgAN, expression of total and phosphor Syk was evaluated in human renal biopsies by immunohistochemistry. Syk (total) was detected on renal tubules, but not in glomeruli of normal kidney tissue from patient with renal carcinoma. The expression level of total Syk was markedly increased in renal tubular cells and slightly increased in glomeruli from patients with IgAN compared to normal control. Similarly, the expression level of phosphorylated Syk (phosphor) was significantly upregulated in both renal tubules and glomeruli. Phosphorylated Syk was not detectable in both compartments of normal kidney tissue (Figure 1).

### Syk Inhibitor Suppressed the Activation of PTECs Cultured With IgA-HMC Medium

**Figure 2:**
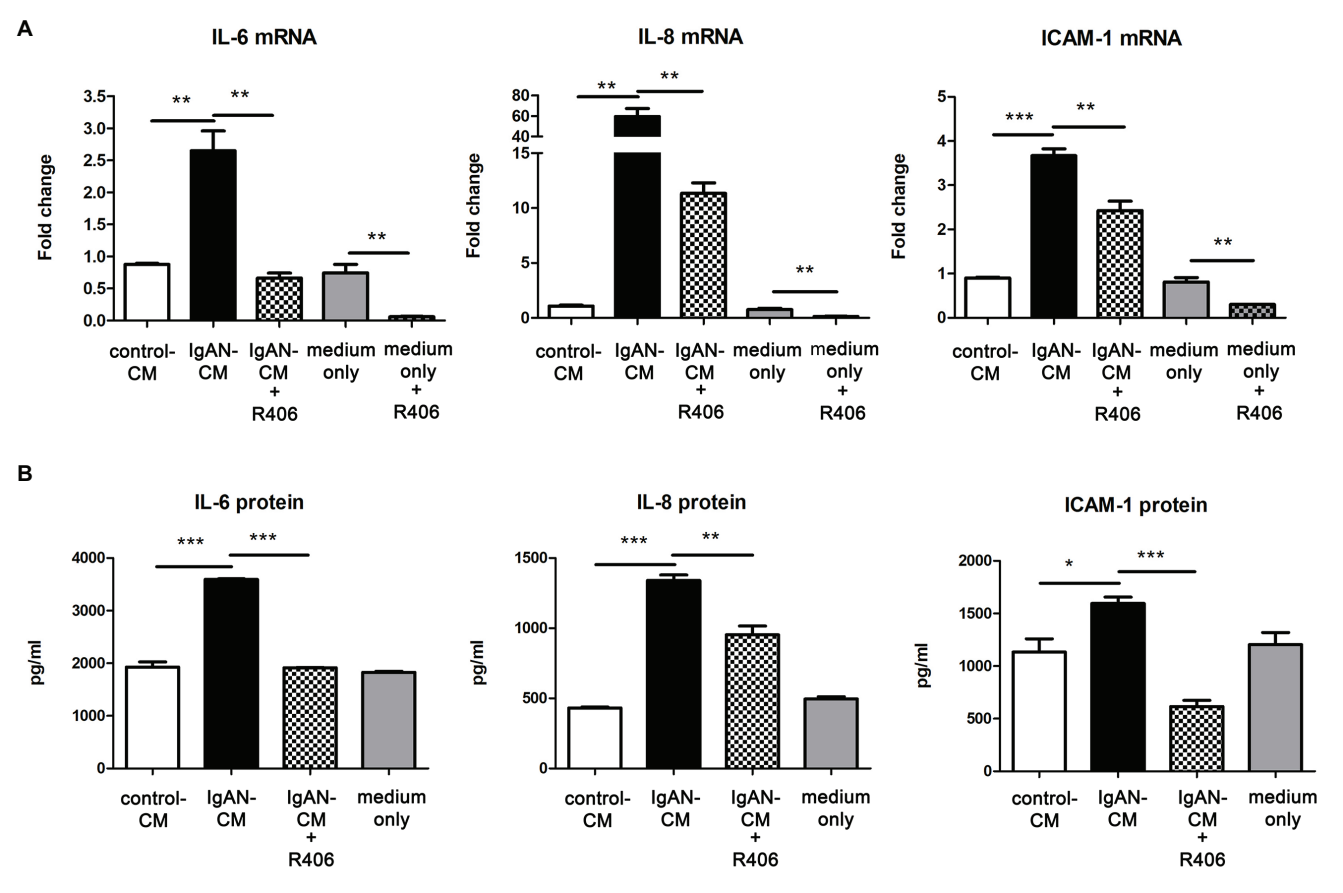
Inhibition of Syk by R406 suppresses inflammatory cytokine expression in proximal tubular epithelial cells (PTECs). **(A)** qPCR and **(B)** ELISA with quantitative analysis on IL-6, IL-8, and ICAM-1 expression in PTECs incubated with conditioned medium from patients with IgAN (IgAN-CM), conditioned medium from healthy control subjects (control-CM) and medium control. ^*^P < 0.05; ^**^P < 0.01 and ^***^P < 0.001 between groups as indicated.

Following the immunohistochemical findings of increased Syk expression levels in renal tubules of IgAN, we next determined whether tubulointerstitial inflammation in IgAN was mediated *via* Syk activation. Cultured PTECs were pretreated with or without Syk inhibitor R406 before incubation with IgA-HMC medium. The expression of IL-6, IL-8, and ICAM-1 mRNA were significantly upregulated in PTECs incubated with conditioned medium from patients with IgAN compared with conditioned medium from healthy control and medium control. Pretreatment with R406 significantly suppressed IgA-HMC medium-induced cytokine expressions from PTECs. Similar inhibitory effect of R406 was observed when PTECs were incubated with medium alone (Figure 2A). Likewise, IL-6, IL-8, and ICAM-1 synthesis was upregulated when PTECs were incubated with IgA-HMC medium and the production of these mediators were attenuated in the presence of R406 (Figure 2B).

### Syk Inhibitor Relieved the Activation of NF-κB and MAPK Signaling PTECs Cultured With IgA-HMC Medium

**Figure 3:**
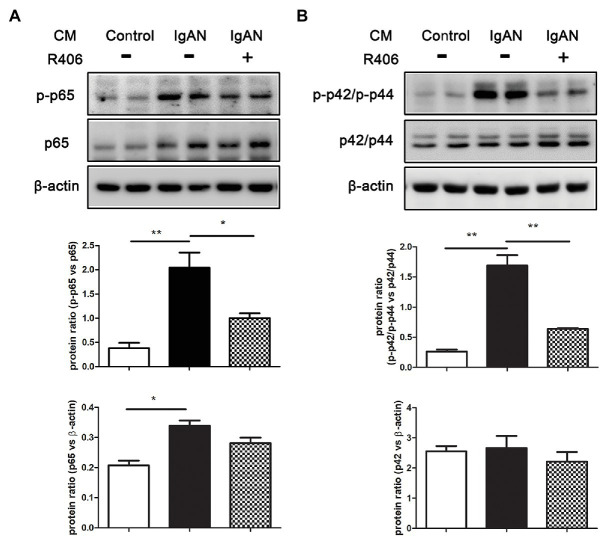
Inhibition of Syk by R406 attenuates activation of NF-κB and MAPK signaling pathway in PTECs. Western blot analysis with quantitative analysis on expression of **(A)** p-p65 and total p65 of NF-κB and **(B)** p-p42/p-p44 and total p42/p44 of MAPK in PTECs incubated with conditioned medium from patients with IgAN (IgAN-CM) with and without R406, and conditioned medium from healthy control subjects (control-CM). β-actin was used as loading control. ^*^p < 0.05 and ^**^p < 0.01 between groups as indicated.

Activation of NF-κB and MAPK signal pathway has been implicated in the pro-inflammatory responses of tubular epithelial cells. We then investigated the effect of Syk inhibition on phosphorylation of p65 NF-κB and p42/p44 MAPK by Western blot analysis. Incubation with IgA-HMC medium from patients with IgAN significantly activated the expression level of phosphorylated p65 (p-p65) and p42/p44 (p-p42/p-p44) in PTECs compared to cells incubated with IgA HMC medium from healthy control. The activation of these signal pathways were markedly suppressed by treatment with Syk inhibitor R406 (Figure 3).

### Syk Activation Was Involved in TNF-α-Induced Pro-inflammatory Cytokine Production in PTECs

**Figure 4:**
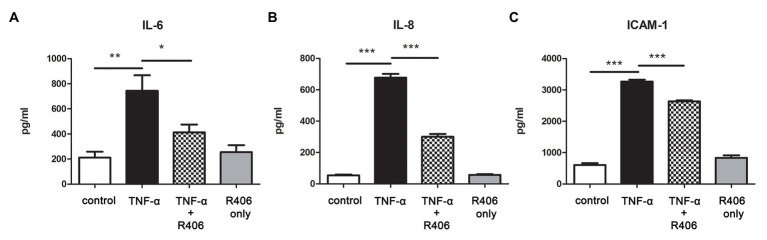
Inhibition of Syk by R406 reduces TNF-α-induced inflammatory cytokine production in PTECs. ELISA with quantitative analysis on protein expression of **(A)** IL-6, **(B)** IL-8 and **(C)** ICAM-1 in culture supernatant from PTECs incubated with control medium, TNF-α and TNF-α pretreated with R406. ^*^p < 0.05; ^**^p < 0.01 and ^***^p < 0.001 between groups as indicated.

Our previous study shows that TNF-α derived from HMC stimulated with IgA from IgAN patients activates tubular cells (Chan et al., 2005b), and it is a potent mediator for NF-κB and p42/p44 MAPK signal transduction. We tested whether Syk activation was involved in TNF-α-induced pro-inflammatory cytokine production in PTECs. In response to TNF-α, synthesis of IL-6, IL-8, and ICAM-1 in supernatant of PTECs was significantly increased as determined by ELISA. Treatment with Syk inhibitor R406 downregulated cytokine synthesis of all three kinds whereas PTECs treated with R406 only had no effect on cytokine synthesis (Figure 4).

## Discussion

This study demonstrates that the expression level of Syk is significantly higher in kidney biopsies from patients with IgAN compared to that from normal control. Moreover, the expression level of phosphorylated Syk is also markedly increased in renal tubules, indicating kinase activation of Syk in tubular cells from patients with IgAN. Of note, increased Syk expression and phosphorylation is observed in the glomeruli from IgAN, which is consistent to the previous study showing the pathogenic role of Syk in IgAN (Kim et al., 2012). Syk is an immunoreceptor-associated signaling protein, and therefore upregulation of phosphorylated Syk confirms the inflammatory events in the tubulointerstitium or activation of tubular epithelial cells in IgAN. Our findings demonstrate that Syk activation not only occurs in glomerular lesions as reported in the previous study, but it also plays a significant role in tubulointerstitial injury.

To confirm our hypothesis that Syk mediates inflammatory responses of activated tubular cells in IgAN, we conducted a pharmacological inhibition study with Syk inhibitor on cultured PTECs. Based on our previous data that tubulointerstitial damage in IgAN is mediated by glomerulotubular communication, *via* humoral factors, rather than direct binding of IgA to PTECs (Chan et al., 2004), cultured PTECs was incubated with conditioned medium from IgA-stimulated HMCs. Syk inhibition by R406 blocks *in vitro* tubular activation by suppressing NF-κB and p42/p44 MAPK signaling pathway and cytokine production in PTECs. R406, the active metabolite of fostamatinib, is a potent Syk inhibitor for blocking of its kinase activity. Numerous studies have demonstrated that R406 reduces immunoreceptor-mediated leukocyte activation and inflammation in the animal model of antibody-induced arthritis (Braselmann et al., 2006), ANCA-associated glomerulonephritis (Mcadoo et al., 2020) and lupus nephritis (Kitai et al., 2017). Syk is important for downstream signal transduction from cell surface immunoreceptor. Our finding shows that IgA-HMC medium prepared from IgAN patients induces activation of NF-κB and p42/p44 MAPK signaling in PTECs, which can be suppressed by Syk inhibitor. Taken together, our data suggest that Syk is required for triggering activation of downstream NF-κB and p42/p44 MAPK pathway in tubular inflammation in IgAN.

Activation of Syk is crucial for regulating intracellular signal transduction in innate immune cells (Lowell, 2011). For example, in human monocytes, tyrosine kinase inhibitor blocks intergrin-mediated Syk phosphorylation and NF-κB-driven IL-β expression (Lin et al., 1995). Other studies have revealed that ligation of C-type lectin receptors in myeloid cells, *via* Syk activation, is coupled to aggregation of caspase-recruitment domain-containing 9 (CARD9) or CARD-containing MAGUK protein 1 (CARMA1) with mucosa-associated lymphoid tissue 1 (Malt1) and B-cell lymphoma 10 (Bcl10), which in turn leads to the activation of downstream NF-κB pathway (Klemm et al., 2006; Hara et al., 2007; Hara and Saito, 2009). Tubular NF-κB expression is correlated with the degree of macrophage infiltration, tubulointerstitial fibrosis and renal survival in renal tissue from patients with primary IgAN (Silva et al., 2011). Thus, activation of tubular epithelial cells is closely associated with renal deterioration. Previous data have found that upregulation of p42/p44 MAPK signaling is detected in activated PTECs *via* glomerulotubular communication. Release of Angiotensin II (Ang II) from IgA-stimulated HMCs binds to Ang II type 1 receptor (AT1R) on PTECs, triggering the phosphorylation of p-42/p44 MAPK and subsequent inflammatory responses (Chan et al., 2005a). Our finding indicates that Syk activation plays a role in p42/p44 MAPK signaling transduction in tubular inflammation in IgAN. However, it is unclear how Syk activation mediates the activation of p42/p44 MAPK signaling pathway. Phosphoinositide 3-kinases (PI3K) have been shown to be a direct binding partner of activated Syk. The Syk–PI3K interaction triggers Akt and PKC signaling pathway, which mediates the activation of p42/p44 MAPK signal transduction (Mocsai et al., 2010). Thus, it is possible that Syk activation mediates downstream p42/p44 MAPK signaling *via* PI3K-Akt-PKC pathway.

In hematopoietic cells, the engagement of immunoreceptor with ITAM triggers Syk activation and the subsequent downstream signal pathways (Turner et al., 2000). In mesangial cells, several potential IgA receptors have been identified for IgA-mediated immune responses. For example, blockade of galactosyltransferase 1 inhibits IgA-induced phosphorylation of Syk and synthesis of IL-6 in HMCs, suggesting IgA binds to galactosyltransferase 1 and leads to Syk activation (Molyneux et al., 2017). However, IgA1 deposits are rarely detected in tubulointerstitium in IgAN and there is no binding of IgA to tubular epithelial cells (Frasca et al., 1982), it remains unclear how Syk is activated in PTECS. Our data show that inhibition of Syk suppresses TNF-α-induced cytokine production in PTECs, suggesting that TNF-α may activate Syk, which in turn induce NF-κB signal transduction. Our previous study demonstrates that TNF-α is a mesangial-derived cytokine that has been implicated in the glomerulotubular crosstalk (Chan et al., 2005b). Indeed, immunoprecipitation study have shown that TNF-α induces the binding of Syk to two TNF-α receptors, TNFR1 and TNFR2, in Jurkat cells and recruitment of Syk modulates TNF-α-induced activation of NF-κB (Takada and Aggarwal, 2004). Taken together, Syk in tubular epithelial cells may be activated by mesangial-derived TNF-α, leading to activation of NF-κB and p42/p44 MAPK signaling pathway.

The importance of Syk activation in the innate immunity is well recognized for decades. Several Syk inhibitors including fostamatinib (R788), entospletinib (GS-9973), cerdulatinib (PRT062070) and TAK-659 are being evaluated under clinical trials (Liu and Mamorska-Dyga, 2017). Accumulating evidence shows that Syk is a crucial player in the pathogenesis of immune-mediated glomerulonephritis such as IgAN, anti-GBM glomerulonephritis and lupus nephritis (Ma et al., 2017). Syk targeting therapy in mouse model of UUO demonstrates a beneficial effect on renal fibrosis (Chen et al., 2016). Treatment with fostamatinib reduces proteinuria and renal inflammation in rats with anti-GBM glomerulonephritis (Chen et al., 2016). Although, we and others have demonstrated the pathogenic role of Syk in mediating inflammatory events in both mesangial cells and tubular epithelial cells, the renoprotective effect of Syk inhibitor has not been investigated in animal model of IgAN due to the lack of a murine line that consistently developing IgAN and the difference in IgA1 between human and rodents. Recent advance in the development of grouped ddY mouse model of IgAN may be a useful tool for future investigation (Suzuki et al., 2014). Furthermore, a phase II randomized controlled trial has been conducted to evaluate the efficacy and safety of fostamatinib in treatment of patients with IgAN (Ma et al., 2016).

In this study, we have demonstrated that Syk is activated in renal tubular cells from patients with IgAN. Inhibition of Syk can suppress inflammatory responses in activated tubular epithelial cells evoked by glomerulotubular crosstalk, *via* downregulation of NF-κB and TNF-α signal transduction. Our findings are in line with previous data on the beneficial effect of Syk inhibition in renal diseases and provide further evidence on the important role of Syk in tubulointerstitial injury and Syk inhibition is a potential therapeutics for IgAN in the future.

## Data Availability Statement

The raw data supporting the conclusions of this article will be made available by the authors, without undue reservation.

## Ethics Statement

The studies involving human participants were reviewed and approved by Research Ethics Committee/Institutional Review Board of the University of Hong Kong/Hospital Authority Hong Kong West Cluster. The patients/participants provided their written informed consent to participate in this study.

## Author Contributions

WY designed and performed the experiment, and wrote the manuscript. KC acquired the clinical specimens and data. LC and JL analyzed and interpreted the results. KL reviewed and edited the manuscript. ST conceived and supervised the study. All authors contributed to the article and approved the submitted version.

### Conflict of Interest

The authors declare that the research was conducted in the absence of any commercial or financial relationships that could be construed as a potential conflict of interest.
